# Detection of mutations in *MYOC*, *OPTN*, *NTF4*, *WDR36* and *CYP1B1* in Chinese juvenile onset open-angle glaucoma using exome sequencing

**DOI:** 10.1038/s41598-018-22337-2

**Published:** 2018-03-14

**Authors:** Chukai Huang, Lijing Xie, Zhenggen Wu, Yingjie Cao, Yuqian Zheng, Chi-Pui Pang, Mingzhi Zhang

**Affiliations:** 1 Joint Shantou International Eye Center of Shantou University and The Chinese University of Hong Kong, Shantou, P. R. China; 2 Department of Ophthalmology and Visual Sciences, The Chinese University of Hong Kong, Hong Kong, P. R. China

## Abstract

Juvenile onset open-angle glaucoma (JOAG) affects patients before 40 years of age, causing high intraocular pressure and severe optic nerve damage. To expand the mutation spectrum of the causative genes in JOAG, with a view to identify novel disease-causing mutations, we investigated *MYOC*, *OPTN*, *NTF4*, *WDR36* and *CYP1B1* in a cohort of 67 unrelated Chinese JOAG patients. Whole exome sequencing was used to identify possible pathogenic mutations, which were further excluded in normal controls. After sequencing and the use of a database pipeline, as well as predictive assessment filtering, we identified a total of six mutations in three genes, *MYOC*, *OPTN* and *CYP1B1*. Among them, 2 heterozygous mutations in *MYOC* (c. 1109C > T, p. (P370L); c. 1150G > C, p. (D384H)), 2 heterozygous mutations in *OPTN* (c. 985A > G, p.(R329G); c. 1481T > G, p. (L494W)) and 2 homozygous mutations in *CYP1B1* (c. 1412T > G, p.(I471S); c. 1169G > A, p.(R390H)) were identified as potentially causative mutations. No mutation was detected in *NTF4* or *WDR36*. Our results enrich the mutation spectra and frequencies of *MYOC*, *OPTN* and *CYP1B1* in JOAG among the Chinese population. Further studies are needed to address the pathogenicity of each of the mutations detected in this study.

## Introduction

Glaucoma, the second leading cause of irreversible blindness worldwide^1^, is a group of heterogeneous optic neuropathies characterized by retinal nerve fibre layer damage and visual field defects. The disease is progressive and leads to permanent visual impairment and even blindness in some patients^2^. Age and high intraocular pressure (IOP) are the main risk factors. Primary open-angle glaucoma (POAG) is a common form of glaucoma, which can be further subdivided into juvenile-onset open-angle glaucoma (JOAG) and adult-onset POAG according to the age of onset^3^. JOAG patients often have higher intraocular pressure (IOP) and suffer from more severe optic nerve damage than adult-onset POAG patients^4^.

Genetic factors play an important role in the development of glaucoma^5^. Several genes have been identified to be associated with POAG, primary congenital glaucoma (PCG) and JOAG, including myocilin (*MYOC*)^6^; optineurin (*OPTN*)^7^; WD repeat domain 36 (*WDR36*)^8^; neurotrophin 4 (*NTF4*)^9^; and cytochrome P450 family 1, subfamily B(*CYP1B1*)^10^. The same candidate gene may lead to different phenotypes of glaucoma^11^. *MYOC* is the first candidate gene mapped for POAG and has been confirmed to be associated with both POAG and JOAG^12^. Mutations of *OPTN* were found in POAG and amyotrophic lateral sclerosis (ALS)^13,14^. *CYP1B1* is a PCG gene but has also been reported in association with JOAG^15,16^. To date, the mutations of known genes only account for approximately 5% of patients with POAG^17^. Compared with adult-onset POAG, JOAG may be more likely to be genetically determined and less likely the consequence of environment^16^. Investigation of the POAG genes in JOAG might provide a good opportunity for understanding the genetic components and heterogeneity of JOAG.

Whole-exome sequencing (WES) is available in commercial service and has been proved to be useful in mapping disease genes. It is rapid and comparatively more cost-effective than other genomic technologies, especially for complex diseases^18^. In developmental and congenital glaucoma, WES has led to the identification of novel variants in LTBP2 and PXDN^19^. It has also been used to identify mutations in known genes in primary glaucoma effectively and quickly^20^. In the current study, we performed WES on 67 Chinese JOAG patients to detect the full spectra of variants in *MYOC*, *OPTN*, *NTF4*, *WDR36* and *CYP1B1*, with a view to identify novel disease-causing mutations for JOAG.

## Results

**Figure 1:**
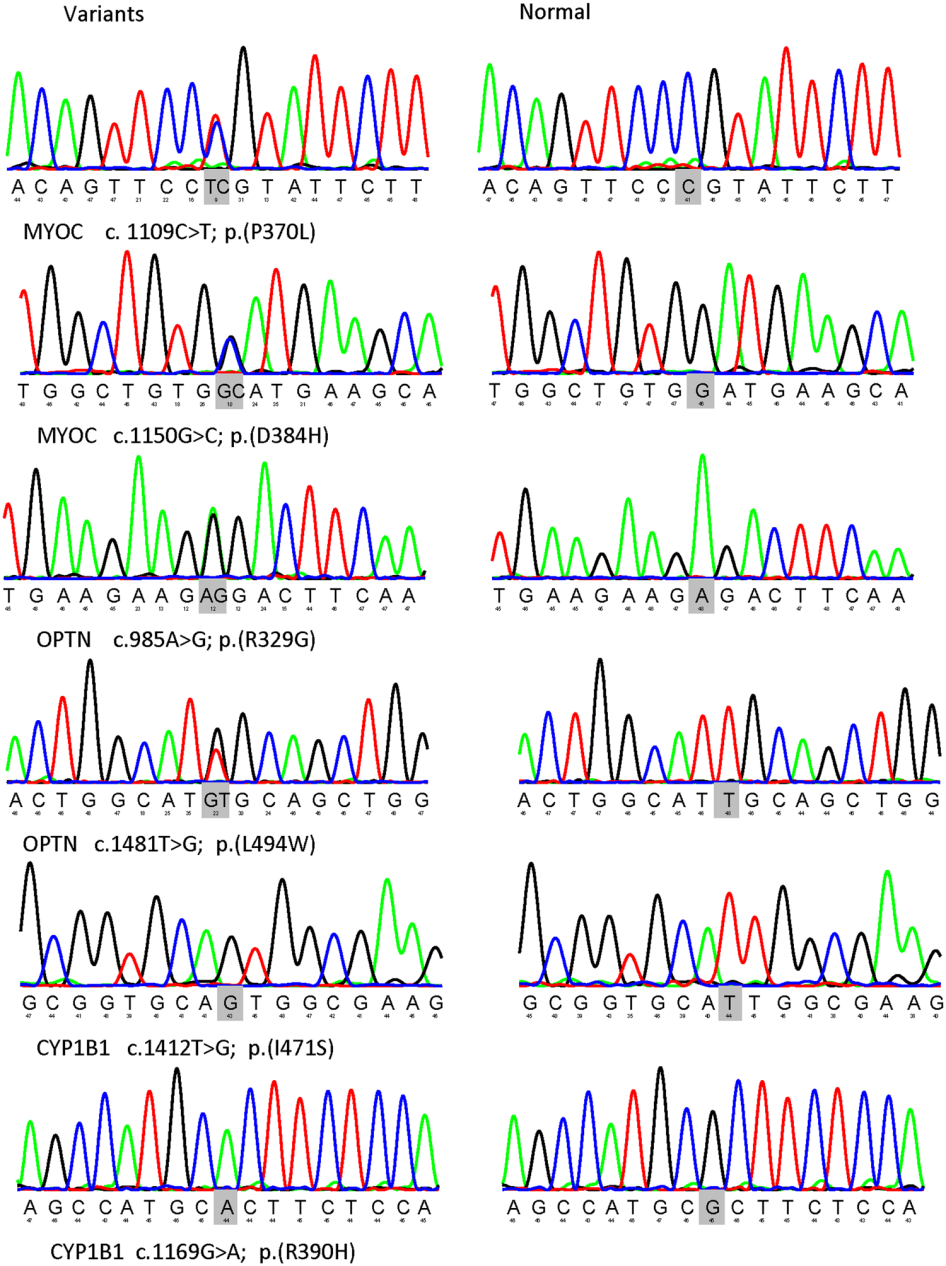
Six variants of *MYOC*, *OPTN* and *CYP1B1* identified in this JOAG cohort. Sequence changes detected in the patients with JOAG are presented in the left column, whereas sequences from healthy individuals appear in the right column.

From the whole exome results of 67 Chinese JOAG patients, totally 79 variants in *MYOC*, 354 variants in *OPTN*, 139 variants in *WDR36*, 45 variants in *NTF4* and 199 variants in *CYP1B1* were detected. Among them, a total of 6 variants in *MYOC*, *OPTN* and *CYP1B1* were identified as potential disease-causing mutations in 8 patients (11.94%) after a series of filtering steps (Tables 1 and 2). These variants included 2 variants in *MYOC*, 2 variants in *OPTN* and 2 variants in *CYP1B1* (Fig. 1). The variants were confirmed by Sanger sequencing. No *MYOC* or *OPTN* potential disease causing mutations were detected in 125 controls, while two heterozygous mutations of *CYP1B1* (c. 1169G > A p. (R390H)) were found in controls (Table 1).

**Table 1:** Mutations of *MYOC*, *OPTN* and *CYP1B1* identified in this study.

Gene	Mutation	Status	Polyphen2	SIFT	Mutation taster	Reported or not	Frequency in control
*MYOC*	c.1109C > T p. (P370L)	Het	D (1)	D (0.02)	DC (0.999)	Reported^26^	0/125
*MYOC*	c.1150G > C p. (D384H)	Het	D (1)	D (0)	DC (0.999)	Novel	0/125
*OPTN*	c.1481T > G p. (L494W)	Het	D (0.999)	D (0.03)	P (0.712)	Reported in ALS^33^	0/125
*OPTN*	c.985A > G p. (R329G)	Het	P (0.856)	T (0.17)	DC (0.519)	Novel	0/125
*CYP1B1*	c.1412T > G p. (I471S)	Hom	D (1)	D (0)	DC (0.999)	Reported^21^	0/125
*CYP1B1*	c.1169G > A p. (R390H)	Hom	D (1)	D (0)	DC (0.999)	Reported^38^	2/125 (Het)

Het, heterozygous mutation; D, damaging; DC, disease causing; P, probably damaging; ALS, Amyotrophic lateral sclerosis; T, tolerated; Hom, homozygous mutation; Reported or not: Mutations with reference citations were reported to be pathogenic.

**Table 2:** Clinical data of the eight patients with mutations.

Case ID	Gene	Mutation	Status	Effect	Age of diagnosis (Y)	Sex	IOP	C/D	VF(MD)
							OD OS	OD OS	OD OS
G303	*MYOC*	c.1109C > T p. (P370L)	hetero	Missense	24	M	54 49	0.9 1.0	−29.36 −34.12
G022	*MYOC*	c.1109C > T p. (P370L)	hetero	Missense	21	M	40 40	1.0 1.0	NA NA
G8–1	*MYOC*	c.1109C > T p. (P370L)	hetero	Missense	23	M	42.3 42.9	0.9 0.9	−32.28 −32.34
G13–1	*MYOC*	c.1150 G > C p. (D384H)	hetero	Missense	25	M	38 33.6	0.9 0.6	−20.28 −2.88
G335	*OPTN*	c.1481T > G p. (L494W)	hetero	Missense	33	F	26 19	0.9 0.4	−29.29 −0.84
G092	*OPTN*	c.985A > G p. (R329G)	hetero	Missense	27	M	31 18	1.0 0.6	−29.88 −0.77
G398	*CYP1B1*	c.1412T > G p. (I471S)	homo	Missense	19	M	47 36	0.9 0.9	−25.78 −32.17
G447	*CYP1B1*	c.1169G > A p. (R390H)	homo	Missense	29	F	38 17	0.9 0.9	−33.54 −23.84

Note: IOP, intraocular pressure; C/D, cup/disc ratio; VF, visual field; MD, mean defect; hetero, heterozygous mutation; homo, homozygous mutation.

**Figure 2:**
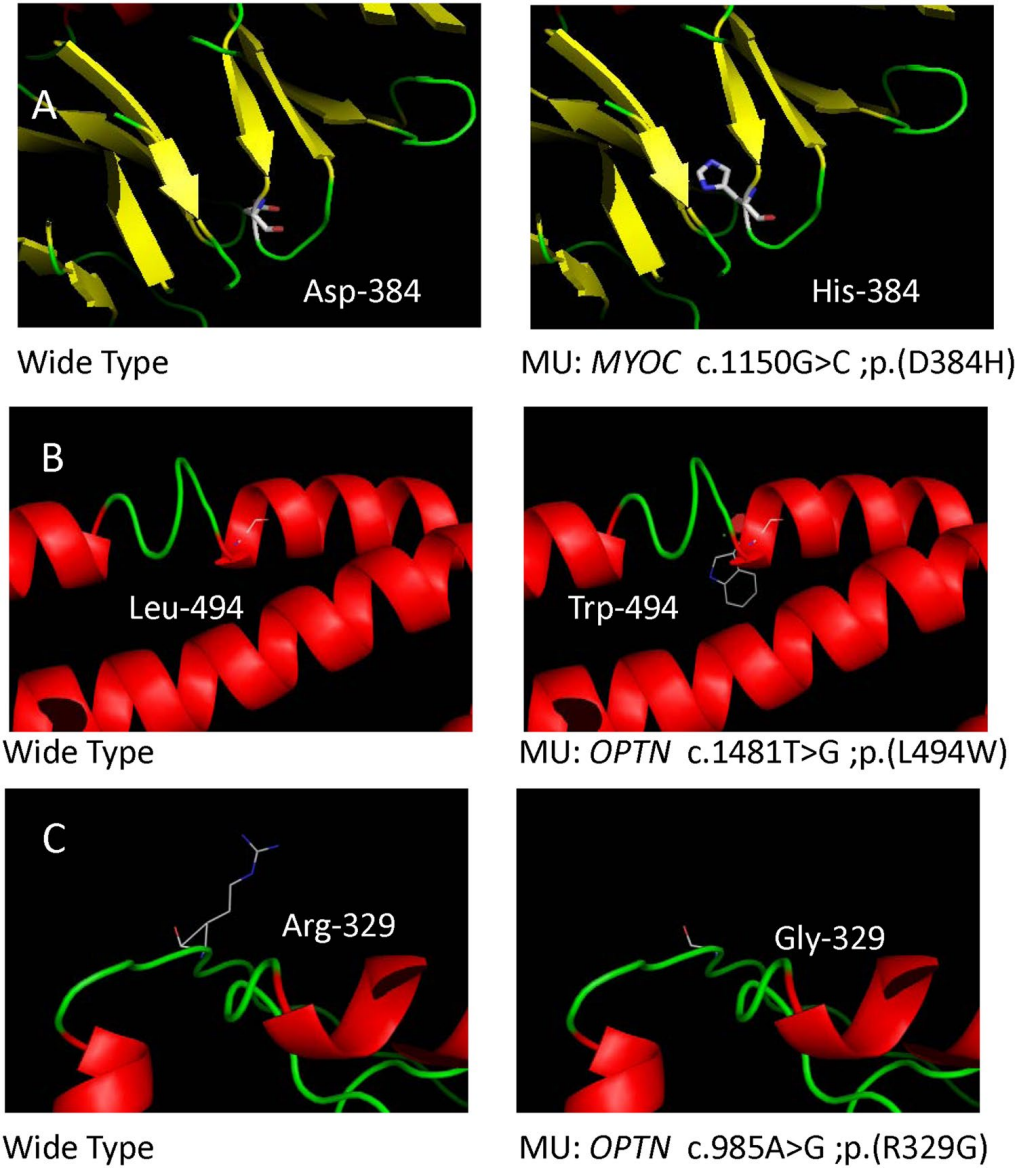
Prediction of the three-dimensional structure of proteins of *MYOC* and *OPTN*. Predicted crystal structures of wild-type (left) and mutant (right) proteins (**A–C**).

**Figure 3:**
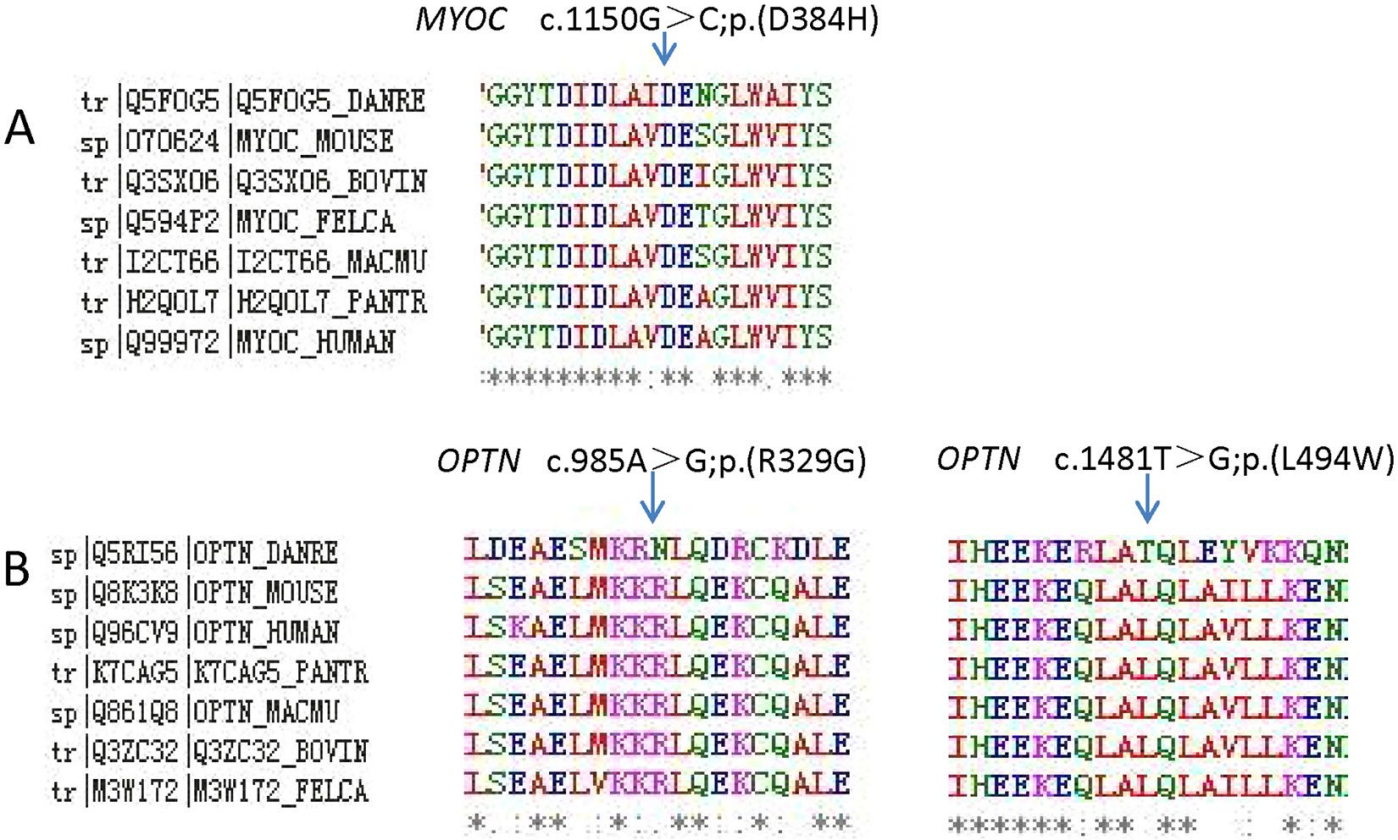
Conservation analysis revealed evolutionary conservation of the mutations by using Clustal Omega. Multiple alignments of the amino acids from different species are shown. The arrow indicates the position of the mutations (**A–C**).

**In** ***MYOC***, there were two heterozygous mutations (c. 1109C > T, p. (P370L); c. 1150G > C, p. (D384H)) detected from two cases in this study, among which p.D384H was novel (Fig. 2A). Substitution of p.D384H was predicted to affect protein function by Polyphen-2, SIFT and Mutation Taster. Additionally, the mutated amino acid is highly conserved among all the tested species (Fig. 3A). p. (P370L) was a reported mutation associated with POAG.

**In** ***OPTN***, 2 heterozygous mutations (c. 985A > G, p. (R329G); c.1481T > G, p. (L494W)) were detected in two unrelated individual. Among them, p. (R329G) was novel and p. (L494W) was reported in amyotrophic lateral sclerosis (ALS) (Fig. 2B,C). p. (R329G) was predicted to be disease causing by Mutation taster and probably damaging by Polyphen-2. Furthermore, the p. (R329G) mutation occurred at a remarkably conserved region in all the tested species apart from danio (Fig. 3B).

**In** ***CYP1B1***, 2 homozygous mutations (c.1412T > G, p. (I471S); c.1169G > A, p. (R390H)) were detected in two patients. Both mutations were predicted to be a pathogenic mutation in all three pathogenicity prediction tools used in this study. The I471S mutation was reported in a Chinese primary congenital glaucoma (PCG) study^21^.

## Discussion

In this study, the results of exome sequencing data from 67 JOAG patients identified 6 mutations in the *MYOC*, *OPTN* and *CYP1B1* genes in 8 unrelated patients. Among them, D384H in *MYOC* and R329G and L494W in *OPTN* were novel. These mutations are predicted to affect protein function and were absent in 125 control individuals without glaucoma.

*MYOC* is the first identified glaucoma-causing gene^22^. Over 20 mutations in *MYOC* have been reported, and the frequency of mutation ranged from 10% to 30%^6,23,24^ in study cohorts with the familial trait. In the present study, 5.97% (4/67) of JOAG patients carry a heterozygous mutation in *MYOC*. Three patients have mutant p. (P370L) in the third exon in *MYOC*. It has been reported that the turnover rate of mutant p. (P370L) in *MYOC* fusion proteins was much prolonged compared with wild type^25^. The ubiquitin-proteasome function is compromised and autophagy is induced with mutant p. (P370L) in *MYOC*^25^. Further study showed a causal association between this p. (P370L) mutation of *MYOC* and juvenile glaucoma with goniodysgenesis^26^. Of the two mutations detected in the present study, p. (P370L) has been previously reported^26,27^. p. (D384H) is a novel mutation in the third exon of *MYOC*, near the position of p. (P370L). Further studies are needed to investigate the biological functions of these two mutations.

The reported role of *OPTN* in JOAG remains inconsistent. Previous studies have identified several mutations in *OPTN* associated with POAG^7^, including c.C160G^28^ and p.(Lys322Glu)^29^. On the other hand, several studies have shown an absence of *OPTN* mutations in POAG or JOAG^30–32^. L494W has been reported in a Chinese amyotrophic lateral sclerosis study but not in any glaucoma study^33^. The patient who carries p. (L494W) is 33 years old. After a detailed systemic review, we confirmed that she has no manifestation f ALS. The mean age at onset of ALS range from 52.9 to 59.9 years in several reports^33,34^. Therefore, it is necessary to follow that patient at regular intervals. p. (R329G) is a novel mutation in *OPTN* in our study. Further studies are needed to understand the role of mutations in *OPTN* in JOAG.

*CYP1B1* is associated with PCG, characterized by an autosomal recessive model. *CYP1B1* is also involved in the development of JOAG. *CYP1B1* (G61E, R368H, R390H, E229K, and 4340delG) may be associated with severe or moderate angle abnormalities and plays an important role in PCG^35^. Suri, F., R, *et al*. first reported that mutations in *CYP1B1* were implicated in POAG among Iranians, notably in the juvenile-onset form^36^. Suri, F., R, *et al*. further reported that PCG nonpenetrant individuals harbouring *CYP1B1* mutations may develop JOAG or POAG to varying degrees^37^. The c.1169 G > A, p. (Arg390His) mutation of *CYP1B1* may be a risk factor for the development of JOAG^38^. In the current study, a homozygous mutation of R390H in the third exon of *CYP1B1* was found in this Chinese JOAG group, consistent with previous reports^38^. A homozygous mutation of I471S in the third exon of *CYP1B1* was identified. The locations of Arg390 and I470 at *CYP1B1* are in the K helix and L helix, respectively. Both helixes are conserved regions for *CYP1B1* and are expected to be involved in proper folding of the molecule. p. (I471S) was first reported to be associated with PCG in a Chinese PCG study^21^, indicating that same genotype may have a different degree of phenotypic expression in Chinese PCG and JOAG. López-Garrido *et al*. have found that the onset of glaucoma in mutant *CYP1B1* genotypes may vary even when *CYP1B1* activity is completely absent. Residual *CYP1B1* activity levels can influence the phenotypic outcome of both homozygous and compound heterozygous carriers, leading to either congenital or non-dominant juvenile glaucoma^39^. These findings help explain why the two homozygous mutations of *CYP1B1* found in our study led to the development of JOAG but not PCG. Further studies are needed to elucidate the role of mutations in *CYP1B1* in the development of JOAG in Chinese patients.

No mutations were detected in *NTF4* or*WDR36*. The role of *NTF4* in POAG remains controversial. *NTF4* is not found to be candidate gene for glaucoma in several studies^40,41^. However, the results of Chen *et al*., Pasutto *et al*. and Vithana *et al*. supported its implication in POAG^42–44^. The mutation frequency of *NTF4* is known to be low in the Chinese population^42,44^. Thus, it is not surprising that no mutation was detected in our small cohort. Mutations of *WDR36* have been detected in different JOAG populations, including German and Chinese^45,46^. The frequency of mutation of *WDR36* is unclear in the Chinese population, and further studies are needed to estimate the *WDR36* mutation frequency in this population.

This study is limited by the lack of parents’ samples so that we are not able to perform segregation analysis. The mutations carriers’ parents were reported to be free of glaucoma. Therefore, whether the mutations detected are de novo mutations cannot be confirmed as for now. Also, there could be other factors that might have affected the penetrance of the mutations so that the patients, even carried the mutations, might not have developed the disease. JOAG may belongs to heterogeneity with different variants in numerous genes, which makes it some familial but most occurring sporadically.

In summary, six variants of *MYOC*, *OPTN* and *CYP1B1* were found in 11.94% of this Chinese JOAG cohort, including two heterozygous mutations in *MYOC* in four patients, two heterozygous mutations in *OPTN* in two patients, and two homozygous mutations in *CYP1B1* in two patients. No mutation of *NTF4* or *WDR36* was detected in this cohort. Although the sample size of this study is small and we have not conducted segregation analysis or functional analyses of the novel mutations, our results provide additional evidence of the mutation spectra and frequencies in Chinese JOAG.

## Materials and Methods

### Patient recruitment

We recruited unrelated Chinese JOAG patients at the Shantou University/Chinese University of Hong Kong Joint Shantou International Eye Center, Shantou, Guangdong Province, China. Written informed consent was obtained from the participants or their guardians. The study was conducted following the tenets of the Declaration of Helsinki and was approved by the institutional review board and ethics committee of Joint Shantou International Eye Center.

The inclusion criteria for patients with JOAG are based on an age of onset between 3 years of age and early adulthood and the manifestation of highly elevated intraocular pressures without angle abnormalities^3^. Briefly, the recruited patients have an onset of open-angle glaucoma between 3 to 40 years old, an intraocular pressure elevated greater than 22 mmHg, characteristic optic disc damage and/or visual field damage, and open angles under gonioscopy. Patients recruited in this study are sporadic JOAG. Blood samples from patients’ parents or siblings were not available. The control subjects were recruited from patients with mild cataracts and age 60 years or above who attended an ophthalmic check-up. The control subjects did not have ophthalmic or systemic diseases.

### Mutational screening

Total genomic DNA was extracted from peripheral blood using a DNA Extraction Kit (QIAGEN, QIAamp® DNA Blood Mini Kit) according to the manufacturer’s instructions. DNA was quantified with Nanodrop 1000 (ND-1000 3.1.0, NanoDrop Spectrophotometer).

WES was performed with an Agilent Sure Select All Human Exon v5.0 kit (Santa Clara, US.). DNA fragments were sequenced using an Illumina HisSeq. 4000 system (Illumina, San Diego, CA). The average sequencing depth was 100-fold. The results were mapped against UCSC hg19 by Burrows-Wheeler Aligner.

Exome sequencing results were filtered with the following steps: (1) Noncoding variants without altering splicing sites predicted by the Berkeley Drosophila Genome Project (available in the public domain at http://www.fruitfly.org/seq_tools/splice.html) were excluded; (2) The synonymous variants without altering splicing sites were removed; (3) SNPs with minor allele frequency (MAF) greater than or equal to 1% in the 1000Genome database were excluded; (4) Missense variants predicted to be benign on protein function consistently by Polyphen-2 (http://genetics.bwh.harvard.edu/pph/), SIFT (availablehttp://sift.jcvi.org)and Mutation Taster (http://www.mutationtaster.org/) were removed. Detected variants affecting coding residues in *MYOC*, *OPTN*, *NTF4*, *WDR36* and *CYP1B1* were selected for further validation and analysis.

Sanger sequencing was used to confirm the candidate variants after filtering. Primers were designed using the Primer3 online tool (Table 3). The methods used to perform Sanger sequencing, including amplification, sequencing, and analysis of the target fragments, have been previously described^47^. Sequence alignment and analysis of variations were performed by using the NovoSNP program^48^.

**Table 3:** Primers used to amplify the sequences harbouring the variants in this study.

Primer Name	Forward	Reverse
*CYP1B*1_3	AGTCATGCAAGGCCTATTACAG	CCACTACTCATGAAGAACCGC
*MYOC*_3	ATTTGTCTCCAGGGCTGTCA	GGTGCCACAGATGATGAAGG
*OPTN*_8	GGATTGATTCACCAGCCAGTC	AAGTTCTCCAGTCCCCAACC
*OPTN*_13	CAGCTTGTATCTGCTATCGGA	AGCTCCCACAAGTCTCTGTC

Note: PCR conditions: 35 cycles of amplification. Each cycle consists of 30 s denaturation at 94 °C, 60 s annealing ranging from 59.5 °C to 60.9 °C and 1 min extension at 72 °C, with a final extension at 72 °C for 5 min.

### Bioinformatics analysis

Clustal Omega (http://www.ebi.ac.uk/Tools/msa/clustalo/) was used to acquire multiple-sequence alignment of *MYOC* and *OPTN* in different species, including Homo sapiens, Pan troglodytes, Macaca mulatta, Bos taurus, Felis catus, Mus musculus, Gallus gallus and Danio rerio. Crystal structures of mutant and wild-type proteins were evaluated by Phyre248 (http://www.sbg.bio.ic.ac.uk/phyre2/html/page.cgi? id = index)^49^ and further visualized using Pymol Molecular Graphics System (Pymol).
